# Supplementary material for: Emergence of sensory attenuation based upon the free-energy principle
Source: Sci Rep. 2022 Aug 25;12:14542. doi: 10.1038/s41598-022-18207-7 (PMC9411191; doi:10.1038/s41598-022-18207-7)
Supplement: Supplementary file 2 — Supplementary Information 2. [file 41598_2022_18207_MOESM2_ESM.pdf]

Supplementary Information for

Emergence of sensory attenuation based upon the free-energy principle

Hayato Idei*, Wataru Ohata, Yuichi Yamashita, Tetsuya Ogata, and Jun Tani*

*Correspondence to; jun.tani@oist.jp or idei@ncnp.go.jp

This PDF file includes:

Supplementary Figures S1 to S9

Caption for Supplementary Video S1

Other supplementary materials for this manuscript include the following:

Supplementary Video S1

Supplementary Figures:

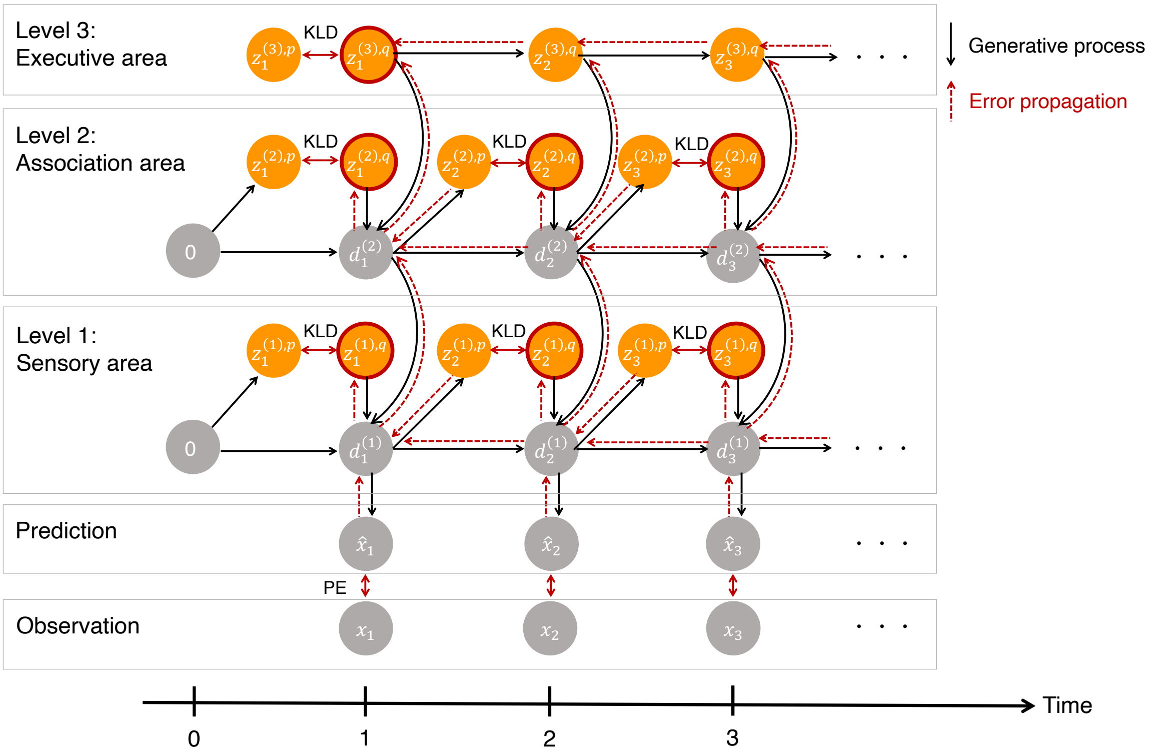

Supplementary Figure S 1: Temporal processing of the recurrent neural network. In the learning phase, the recurrent network updates the posteriors of latent variables in all areas ($\boldsymbol{z}_{1:T}^{(1),q}$, $\boldsymbol{z}_{1:T}^{(2),q}$, and $\boldsymbol{z}_1^{(3),q}$) and time-constant synaptic weights via minimization of free-energy over the time length (T) of training data. Initial deterministic states $\boldsymbol{d}_0$ in all areas are set to 0. The initial prior distribution $\boldsymbol{z}_1^p$ in the executive area is set to a unit Gaussian distribution $\mathcal{N}(0, 1)$. For simplicity, the distributed structure of sensory areas is omitted. PE: Prediction error. KLD: Kullback-Leibler divergence between the posterior and the prior of latent variable.

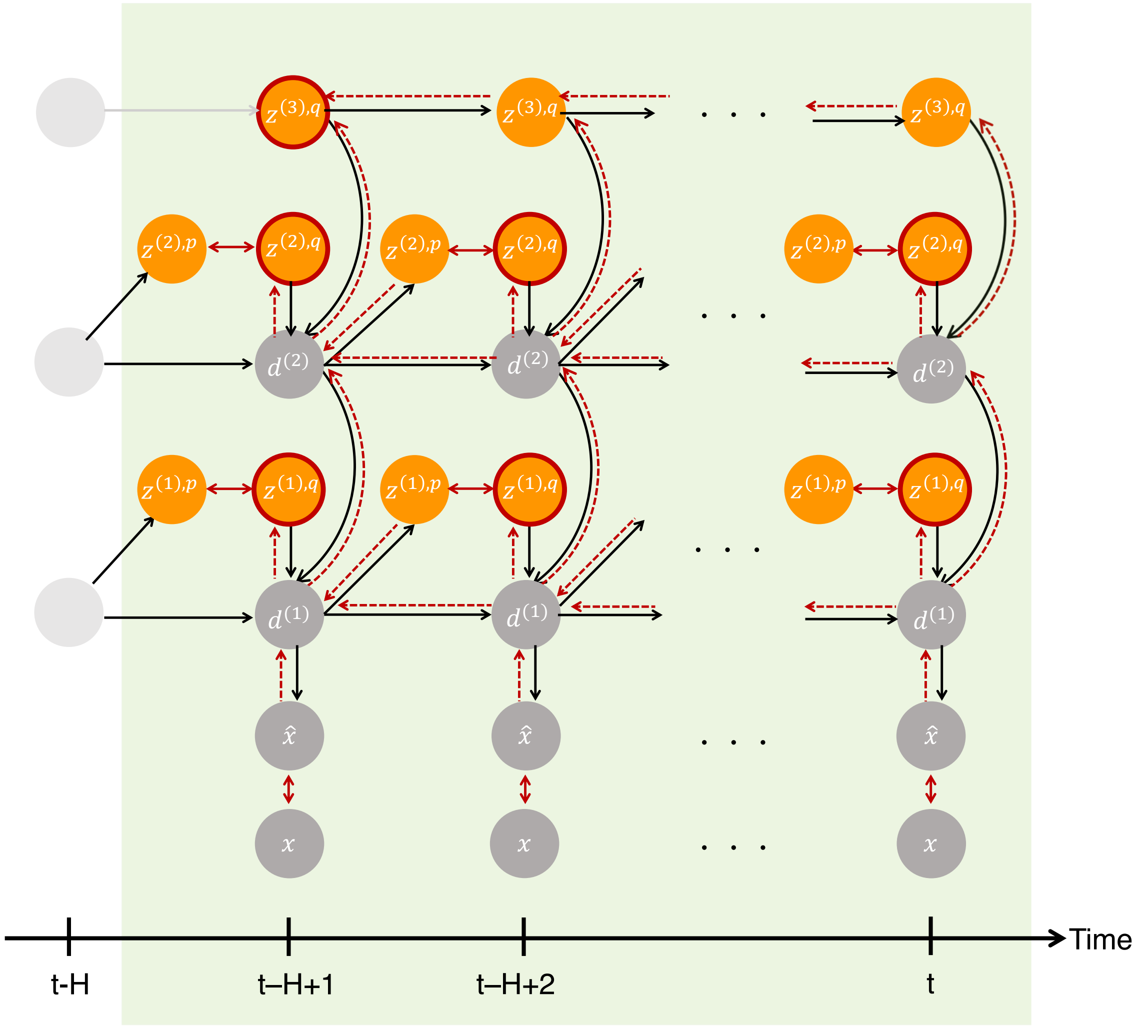

Supplementary Figure S 2: Online inference in the test phase. The recurrent network updates the posterior ($z^{(1),q}_{t-H+1:t}$, $z^{(2),q}_{t-H+1:t}$, and $z^{(3),q}_{t-H+1}$) via minimization of the free-energy over a certain time window (H). Synaptic weights are fixed. For simplicity, the distributed structure of sensory areas is omitted.

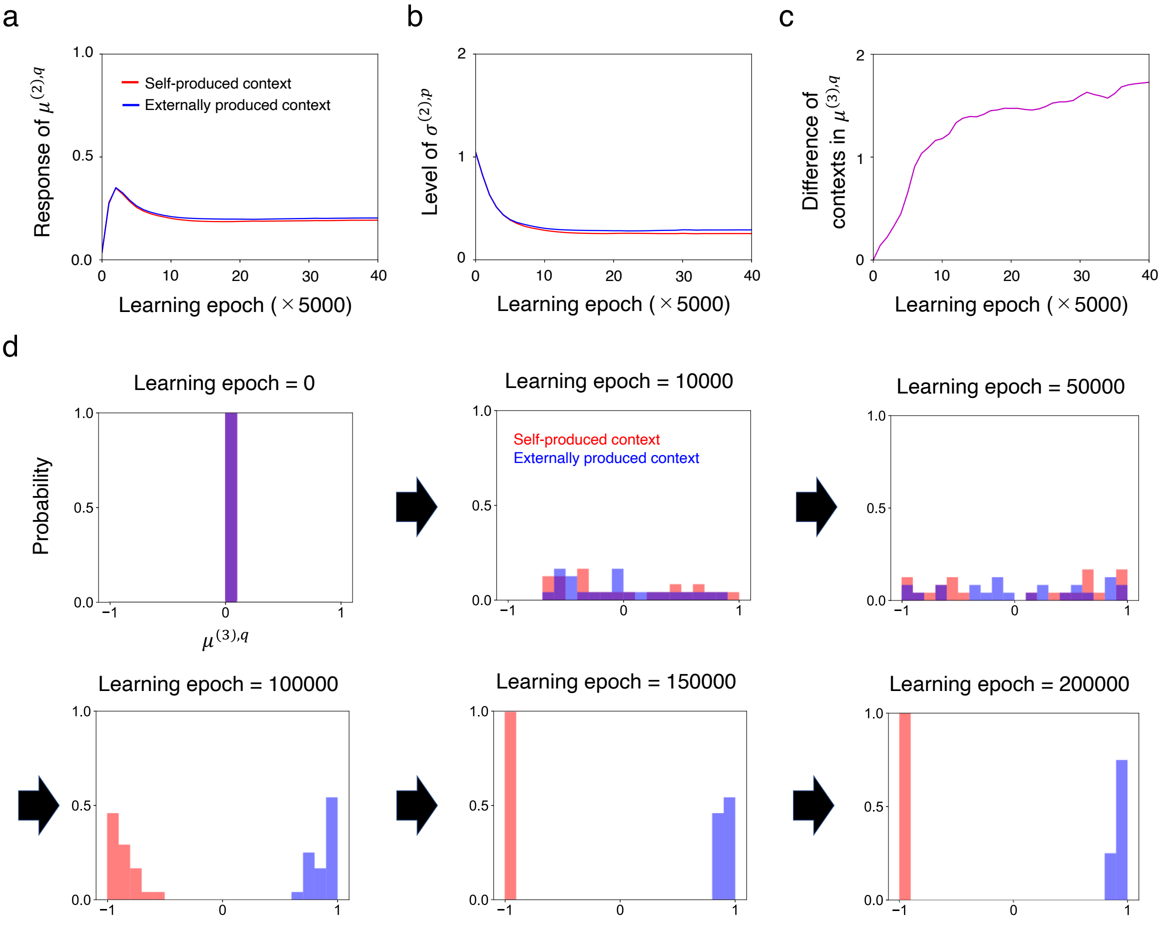

Supplementary Figure S 3: Development of higher-level latent variables. **(a-b)** Development of a posterior response and a prior sigma in the association area. At each learning epoch, the value is averaged over 200 time steps, 24 training datasets, and 10 networks. **(c)** Development of the difference between self-produced contexts and externally produced contexts in the executive area. In the learning phase, the recurrent network developed a posterior state in the executive area for each of 48 training datasets (24 for self-produced contexts and 24 for externally produced contexts). The figure shows the distance between median values of posterior states for self-produced and externally produced contexts. At each learning epoch, the value is averaged over 10 networks. **(d)** An example of development of the executive-level posterior. Each figure shows the distribution of 24 executive-level posterior states corresponding to self-produced or externally produced contexts. The figure shows that recognition of different sensorimotor experiences was gradually developed through a learning process. Note that we did not provide any explicit label indicating the difference between the two contexts.

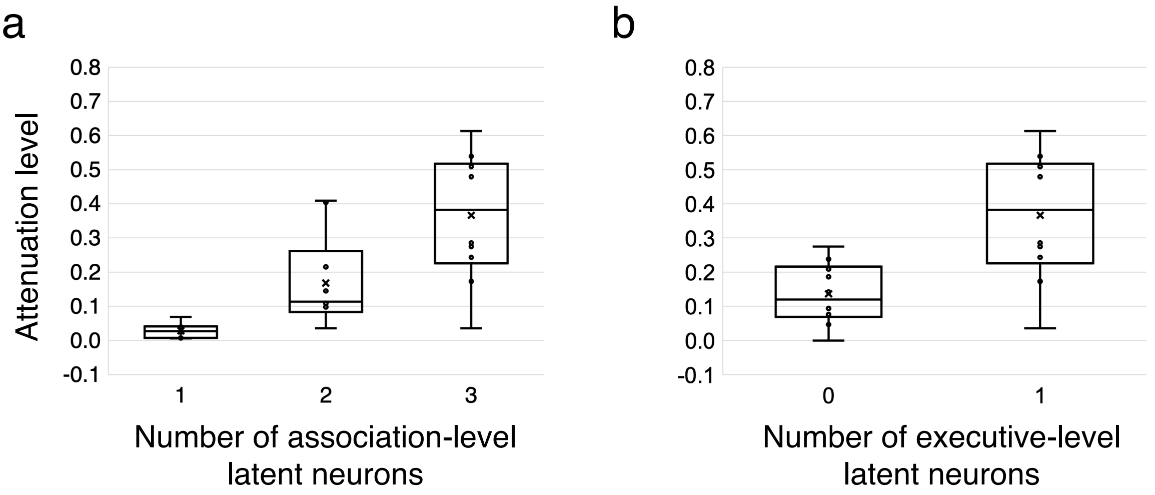

Supplementary Figure S 4: Neural-response attenuation varying with the number of latent neurons. The attenuation level was calculated by subtracting the sensory-level posterior response in a self-produced context from that in an externally produced context while reproducing training data. Values were for 10 trained networks with different initial synaptic weights. **(a)** A larger number of association-level latent neurons led to a larger attenuation level, suggesting that representing sensorimotor correlation required enough association-level latent neurons. **(b)** When there were no executive-level latent neurons, the attenuation level was greatly reduced. This suggests the importance of executive-level control for sensory attenuation. In (a) and (b), the setting of 3 association-level and 1 executive-level latent neurons was the same baseline model.

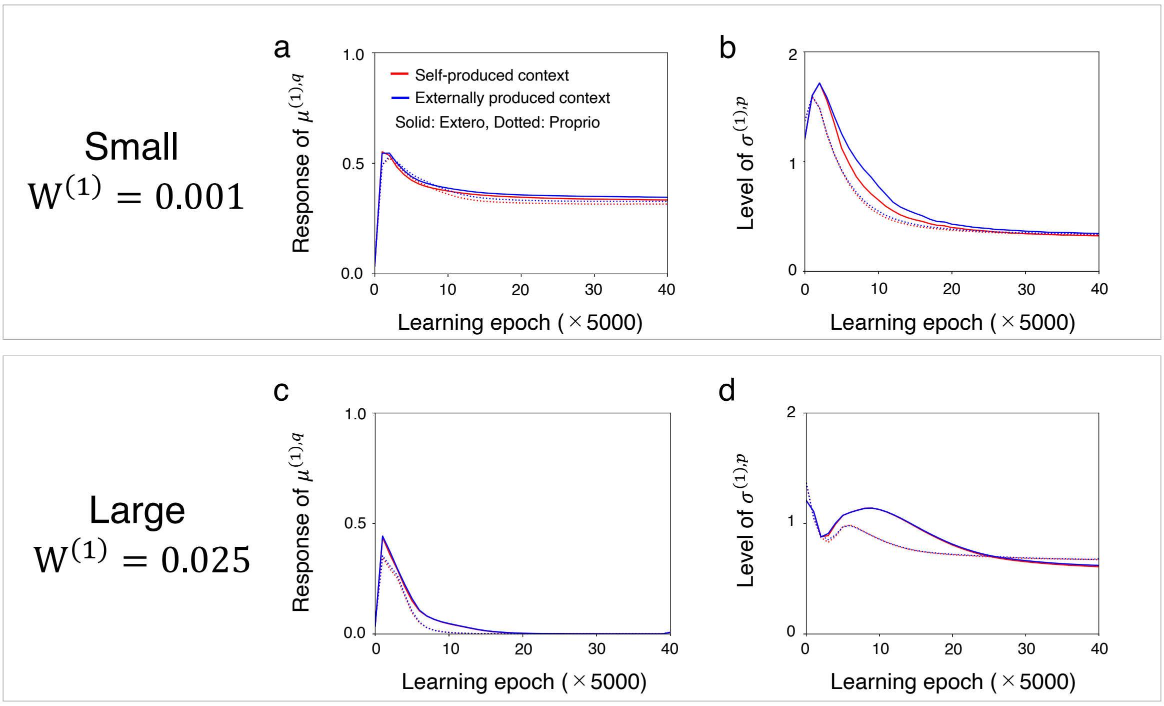

Supplementary Figure S 5: Effects of shifts in the meta-prior in sensory areas. **(a-b)** Development of a sensory-level posterior response and prior sigma through learning in a small sensory-level meta-prior setting ($W^{(1)} = 0.001, W^{(2)} = W^{(3)} = 0.005$), described as in Fig. 4c-d. A small sensory-level meta-prior led to reduced attenuation of the sensory-level posterior response, as well as the sensory-level prior sigma, in the self-produced context. **(c-d)** Development of a sensory-level posterior response and a prior sigma in a large sensory-level meta-prior setting ($W^{(1)} = 0.025, W^{(2)} = W^{(3)} = 0.005$). A large sensory-level meta-prior led to highly reduced sensory-level posterior responses in both self-produced and externally produced contexts.

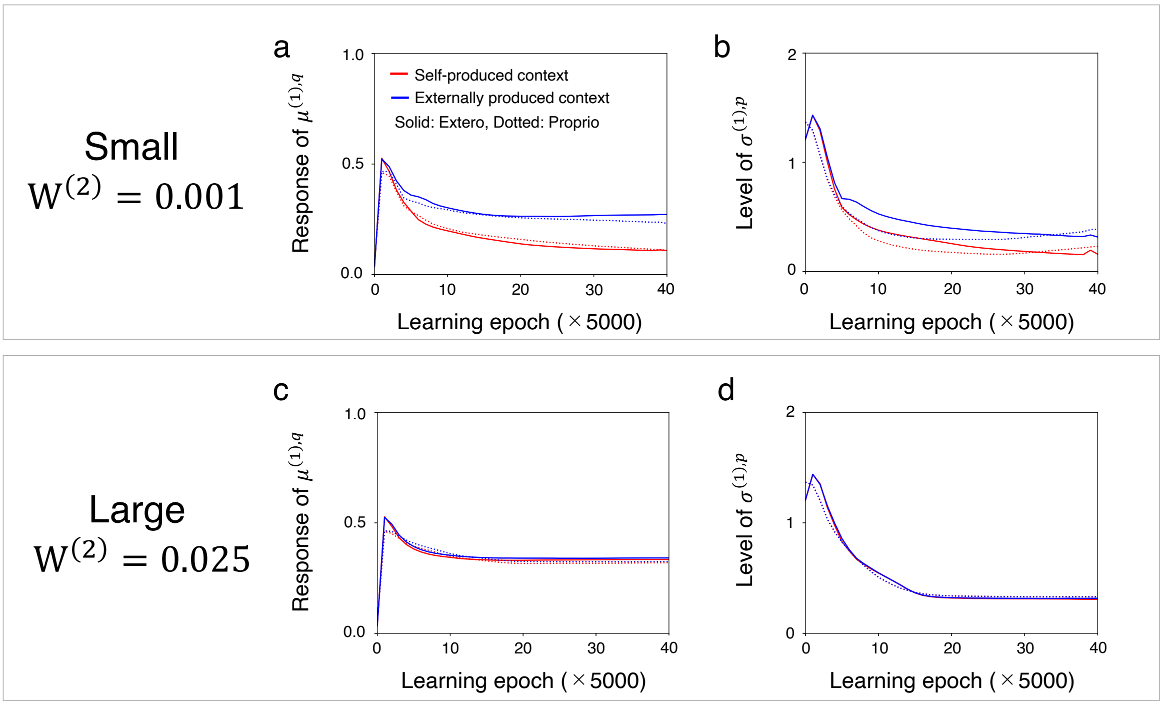

Supplementary Figure S 6: Effects of shifts in the meta-prior in the association area. **(a-b)** Development of a sensory-level posterior response and prior sigma through learning in a small association-level meta-prior setting ($W^{(2)} = 0.001, W^{(1)} = W^{(3)} = 0.005$), described as in Fig. 4c-d. A small association-level meta-prior did not have a large impact on development of sensory attenuation. **(c-d)** Development of a sensory-level posterior response and a prior sigma in a large association-level meta-prior setting ($W^{(2)} = 0.025, W^{(1)} = W^{(3)} = 0.005$). A large association-level meta-prior led to reduced attenuation of the sensory-level posterior response, as well as the sensory-level prior sigma, in the self-produced context.

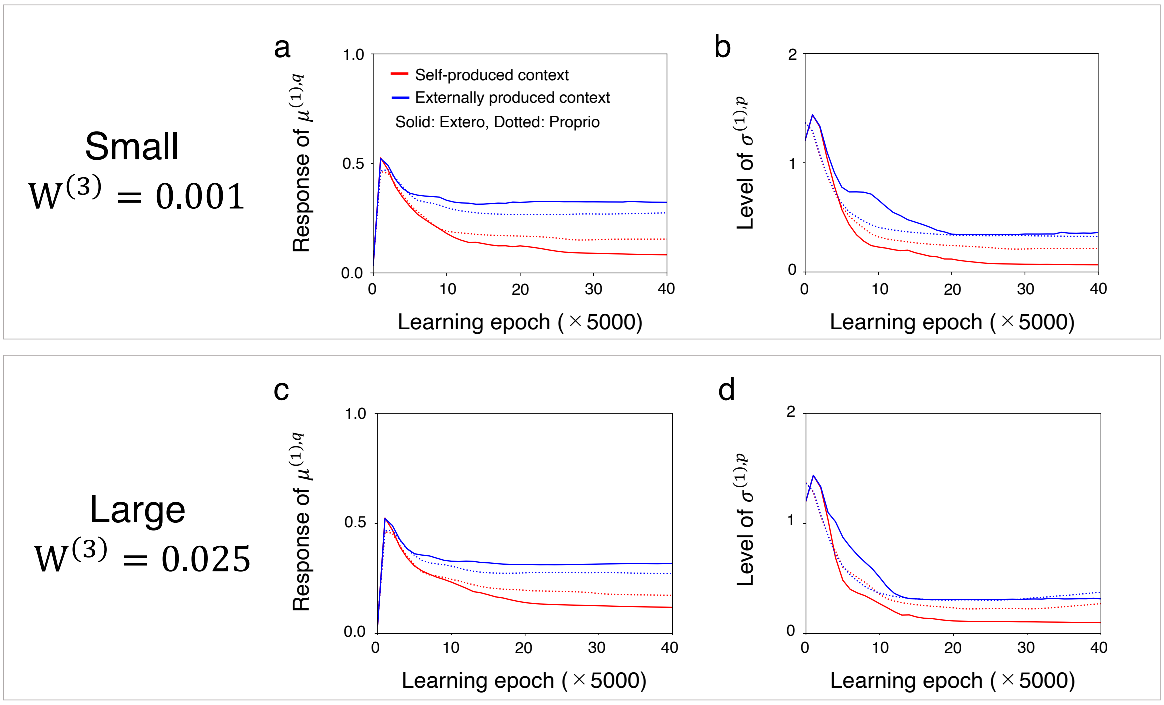

Supplementary Figure S 7: Effects of shifts in the meta-prior in the executive area. **(a-b)**Development of a sensory-level posterior response and prior sigma through learning in a small executive-level meta-prior setting ($W^{(3)} = 0.001, W^{(1)} = W^{(2)} = 0.005$), described as in Fig. 4c-d. **(c-d)** Development of a sensory-level posterior response and a prior sigma in a large executive-level meta-prior setting ($W^{(3)} = 0.025, W^{(1)} = W^{(2)} = 0.005$). Neither small nor large executive-level meta-priors had a large impact on development of the sensory-level posterior response or the prior sigma.

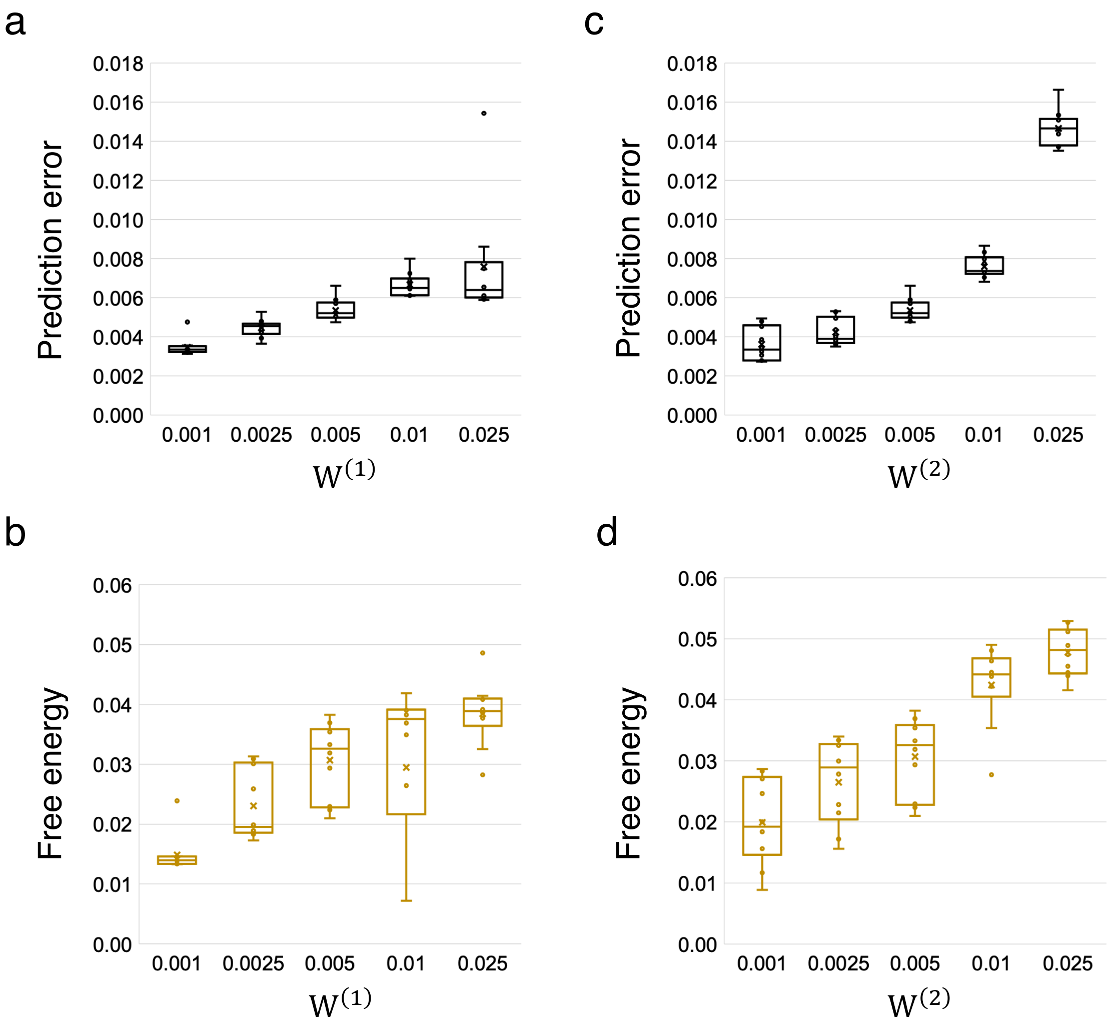

Supplementary Figure S 8: Training results. **(a-b)** Prediction error and free-energy for training data under each meta-prior setting in sensory areas ($W^{(2)} = W^{(3)} = 0.005$). A small sensory-level meta-prior led to decreases in the prediction error and free-energy, although development of sensory attenuation was disrupted. **(c-d)** The prediction error and free-energy for training data under each meta-prior setting in the association area ($W^{(1)} = W^{(3)} = 0.005$). In (a)-(d), the setting of $W^{(1)} = W^{(2)} = W^{(3)} = 0.005$ was the same baseline model.

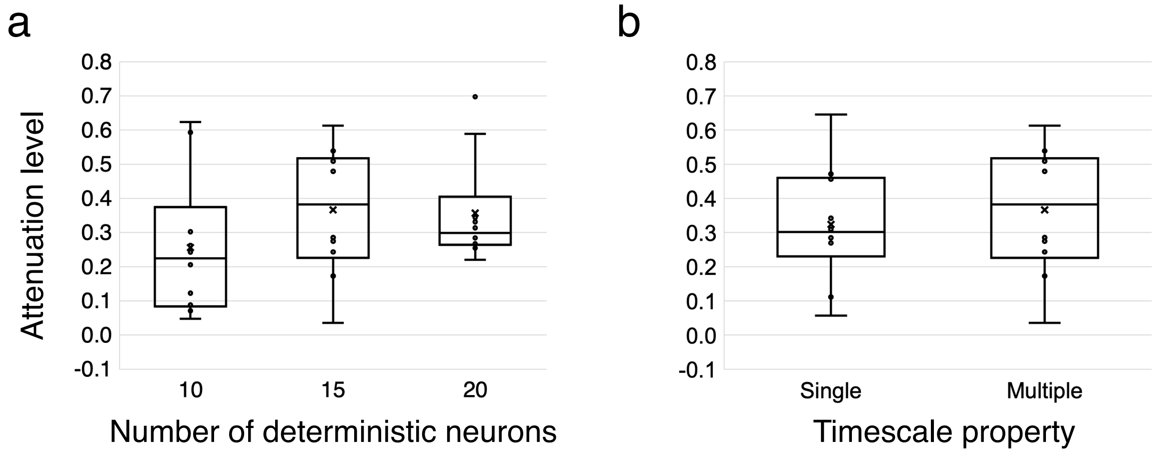

Supplementary Figure S 9: Neural-response attenuation varied with settings of deterministic neurons. The attenuation level was calculated by subtracting the sensory-level posterior response in the self-produced context from that in the externally produced context while reproducing training data. Values were for 10 trained networks with different initial synaptic weights. **(a)** A recurrent network with 15 deterministic neurons showed the largest average (and median) attenuation level. **(b)** Multiple timescale settings ($\tau = 2$ for 8 neurons and $\tau = 4$ for 7 neurons) increased the attenuation level compared to the single timescale setting ($\tau = 2$ for all deterministic neurons). In (a) and (b), the setting of 15 deterministic neurons and multiple timescale property was the same as the baseline model.

Caption for Supplementary Video:

Supplementary Video S1. Robot behavior and neural responses in the baseline model. Related to Fig. 2b-e. Red and blue crosses in the left image indicate predicted positions of the external object and robot hand, respectively.
